# Mycetoma trichophyton

**DOI:** 10.11604/pamj.2021.40.121.32006

**Published:** 2021-10-27

**Authors:** Evangelia Christodoulou, Savas Deftereos

**Affiliations:** 1 Democritus University of Thrace, Medical School, University Campus Dragana, Alexandroupolis, Greece,; 2 Department of Radiology, University General Hospital of Alexandroupolis, Alexandroupolis, Greece

**Keywords:** Mycetoma, trichophyton, ultrasound, X-ray, magnetic resonance imaging, pediatric, radiology

## Image in medicine

A 2-year-old female child admitted to the hospital due to a palpable mass at her scalp referred by her parents. Her past medical history was clear. The clinical examination revealed edema, redness/erythema, blisters, tenderness and itching. No neurological changes were referred, no hair loss occurred and no other areas of the body were affected. The initial evaluation included X-rays, an ultrasonography and a magnetic resonance imaging (MRI) scan. The imaging findings were not pathognomonic but in combination with the clinical features, the differential diagnosis shrinked between mycetoma and pilomatrixoma (or calcifying epithelioma of Malherbe). For further evaluation, a small specimen of the scalp was obtained by scraping the skin with a scalpel. Cytological investigation revealed fungi infection caused by trichophyton species. So the final accurate diagnosis was mycetoma trichophyton. Patient was treated with miconazole nitrate (daktarin cream 2%), 1x3 for 15 days. First follow-up (day 15) showed improvement to complete cure. At the second follow up (day 30) there was no evidence of relapse. Mycetoma trichophyton must be differentiated from pilomatrixoma (which may appear with similar clinical and sonographic features), cutaneous tuberculosis, folliculitis, actinomycosis, chromomycosis, sporotrichosis, coccidioidomycosis, blastomycosis of the skin, botryomycosis etc. Dermatophyte infections usually occur in immuno compromised patients. In our case, the young patient was healthy and no medication administration had previously happened. A trichophyton infection mainly causes superficial lesions to the skin, nails and hair, deeper dermis and subcutaneous tissue anywhere on the body but most frequently at the lower extremities.

**Figure 1:**
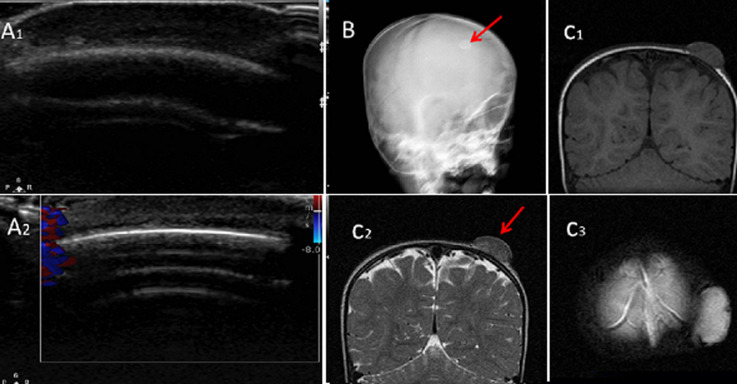
A) (1,2) ultrasound examination findings at the area of interest; B) skull radiography; C) (1,2,3) magnetic resonance imaging (MRI) scan (T1W, T2W and flair sequences)

